# Characterization of Multiple Ion Channels in Cultured Human Cardiac Fibroblasts

**DOI:** 10.1371/journal.pone.0007307

**Published:** 2009-10-06

**Authors:** Gui-Rong Li, Hai-Ying Sun, Jing-Bo Chen, Yuan Zhou, Hung-Fat Tse, Chu-Pak Lau

**Affiliations:** 1 Department of Medicine and Research Centre of Heart, Brain, Hormone and Healthy Aging, Li Ka Shing Faculty of Medicine, The University of Hong Kong, Pokfulam, Hong Kong Special Administrative Region (SAR), China; 2 Department of Physiology, Li Ka Shing Faculty of Medicine, The University of Hong Kong, Pokfulam, Hong Kong Special Administrative Region (SAR), China; Instituto de Química, Universidade de São Paulo, Brazil

## Abstract

**Background:**

Although fibroblast-to-myocyte electrical coupling is experimentally suggested, electrophysiology of cardiac fibroblasts is not as well established as contractile cardiac myocytes. The present study was therefore designed to characterize ion channels in cultured human cardiac fibroblasts.

**Methods and Findings:**

A whole-cell patch voltage clamp technique and RT-PCR were employed to determine ion channels expression and their molecular identities. We found that multiple ion channels were heterogeneously expressed in human cardiac fibroblasts. These include a big conductance Ca^2+^-activated K^+^ current (BK_Ca_) in most (88%) human cardiac fibroblasts, a delayed rectifier K^+^ current (IK_DR_) and a transient outward K^+^ current (I_to_) in a small population (15 and 14%, respectively) of cells, an inwardly-rectifying K^+^ current (I_Kir_) in 24% of cells, and a chloride current (I_Cl_) in 7% of cells under isotonic conditions. In addition, two types of voltage-gated Na^+^ currents (I_Na_) with distinct properties were present in most (61%) human cardiac fibroblasts. One was a slowly inactivated current with a persistent component, sensitive to tetrodotoxin (TTX) inhibition (I_Na.TTX_, IC_50_ = 7.8 nM), the other was a rapidly inactivated current, relatively resistant to TTX (I_Na.TTXR_, IC_50_ = 1.8 µM). RT-PCR revealed the molecular identities (mRNAs) of these ion channels in human cardiac fibroblasts, including KCa.1.1 (responsible for BK_Ca_), Kv1.5, Kv1.6 (responsible for IK_DR_), Kv4.2, Kv4.3 (responsible for I_to_), Kir2.1, Kir2.3 (for I_Kir_), Clnc3 (for I_Cl_), Na_V_1.2, Na_V_1.3, Na_V_1.6, Na_V_1.7 (for I_Na.TTX_), and Na_V_1.5 (for I_Na.TTXR_).

**Conclusions:**

These results provide the first information that multiple ion channels are present in cultured human cardiac fibroblasts, and suggest the potential contribution of these ion channels to fibroblast-myocytes electrical coupling.

## Introduction

It is generally recognized that cardiac myocytes and fibroblasts form extensive networks in the heart, with numerous anatomical contacts between cells [1]. Cardiac fibroblasts play a central role in the maintenance of extra-cellular matrix in the normal heart and act as mediators of inflammatory and fibrotic myocardial remodeling in the injured heart, e.g. ischemic, hypertensive, hypertrophic, and dilated cardiomyopathies, and heart failure [2], [3]. The cardiac myocyte network, coupled with gap junctions, is generally believed to be electrically isolated from fibroblasts in vivo. However, in the co-culture of cardiac myocytes and fibroblasts, the heterogeneous cell types form functional gap junctions, providing a substrate for electrical coupling of distant myocytes, interconnected by fibroblasts. In addition to the evidence of fibroblast-to-myocyte electrical coupling in the rabbit SA node [4], fibroblasts have been shown to be coupled electrotonically with myocytes in vitro [1], [5]–[8]. Moreover, there is increasing evidence that implicates potential heterocellular electrical coupling in the diseased myocardium with arrhythmogenesis [9], [10]; therefore, the cardiac fibroblasts are considered to be potential targets in managing cardiac disorders including hypertrophy, heart failure and arrhythmias [2], [3], [9], [10].

Ion channels and their functions are well studied in cardiomyocytes; however, the ion channel expression and their physiological roles are not fully understood in cardiac fibroblasts. An inward rectifier K^+^ current (I_Kir_), a delayed rectifier K^+^ current (IK_DR_), and a non-selective cation channel current were recently reported in rat ventricular fibroblasts [11]–[13]. Although a Ca^2+^-activated big conductance K^+^ current (BK_Ca_) was described in human cardiac fibroblasts [14], it is unknown whether other types of ion channel currents are present in human cardiac fibroblasts. The present study was designed to employ the approaches of whole-cell patch voltage clamp and RT-PCR to examine the functional ion channels in human cardiac fibroblasts. Using these techniques, we identified multiple ion channels expressed in cultured human cardiac fibroblasts.

## Methods

### Cell cultures

Human cardiac fibroblasts (adult ventrical, Catalog# 6310) were purchased from ScienCell Research Laboratory (San Diego, CA). The cells were cultured as monolayers in completed DMEM containing 10% fetal bovine serum (Invitrogen, Hong Kong) and antibiotics (100 U/ml penicillin G and 100 µg/ml streptomycin) at 37°C in a humidified atmosphere of 95% air, 5% CO_2_. No difference in cell growth and ion channel expression were observed with either our culture medium or the medium from ScienCell Research Laboratory. Cells used in this study were from the early passages 2 to 6 to limit the possible variations in functional ion channel currents and gene expression. The cells were harvested for electrophysiological recording and RT-PCR determination via trypsinization [15].

### Solutions and reagents

Tyrode solution for electrophysiological study contained (mM): 140 NaCl, 5.0 KCl, 1.0 MgCl_2_, 1.8 CaCl_2_, 10 glucose, and 10 HEPES; pH was adjusted to 7.3 with NaOH. The standard pipette solution contained (mM): 20 KCl, 110 K-aspartate, 1.0 MgCl_2_, 10 HEPES, 0.05 EGTA, 0.1 GTP, 5.0 Na_2_-phosphocreatine, and 5.0 Mg-ATP; pH was adjusted to 7.2 with KOH. When Na^+^ current was determined, K^+^ in pipette and bath solutions was replaced by equimolar Cs.

For volume sensitive chloride current (I_Cl.vol_) recording, hypotonic 0.7T (∼210 mosmol/L) Tyrode solution was made by reducing NaCl from 140 to 98 mM. When 1.0T (∼300 mosomol/L) solution was prepared, 90 mM mannitol was added. The pipette solution for recording I_Cl.vol_ contained (mM) 110 CsCl, 20 Cs-aspartate, 5 EGTA, 1.0 MgCl_2_, 10 HEPES, 0.1 GTP, 5.0 Na_2_-phosphocreatine, and 5.0 Mg-ATP (pH = 7.2 with CsOH).

The chloride channel blocker 5-nitro-1-(3-phenylpropylamino) benzoic acid (NPPB) was purchased from Tocris (Bristol, UK). All the other chemicals including DIDS (4,4′-diisothiocyanostilbene-2,2′-disulfonic acid) were purchased from Sigma-Aldrich (St Louis, MO).

### Electrophysiology

A small aliquot of the solution containing the cardiac fibroblasts was placed in an open perfusion chamber (1 ml) mounted on the stage of an inverted microscope. The cells were allowed to adhere to the bottom of the chamber for 10–20 min, and then superfused at 2–3 ml/min with Tyrode solution. The studies were conducted at room temperature (22–24°C).

The membrane ionic currents were recorded with a whole-cell patch-clamp technique as described previously [16]. Borosilicate glass electrodes (1.2 mm OD) were pulled with a Brown–Flaming puller (Model P-97, Sutter Instrument Co. Novato, CA), and had tip resistances of 2∼3 MΩ when filled with pipette solution. The tip potentials were compensated before the pipette touched the cell. After a gigaohm-seal was obtained by negative pressure suction, the cell membrane was ruptured by a gentle suction to establish whole-cell configuration with a seal resistance >800 MΩ. The cell membrane capacitance (49.6±12.1 pF) was electrically compensated with the Pulse software. The series resistance (R_s_, 3–5 MΩ) was compensated by 50–70% to minimize voltage errors. Membrane currents were elicited with voltage protocols as described in the following Results section for individual different current recording. Data were acquired with an EPC10 amplifier (Heka, Lambrecht, Germany). The membrane currents were low-pass filtered at 5 kHz and stored on the hard disk of an IBM compatible computer.

### Messenger RNA determination

The messenger RNA was examined using RT-PCR technique using Table 1 primers as described previously [15]. Total RNA was extracted from human cardiac fibroblasts using Trizol reagent (Invitrogen), and further treated with DNase I (GE Healthcare, Hong Kong) for 30 min at 37°C, then heated to 75°C for 5 min and finally cooled to 4°C [17]. Reverse transcription was performed using a RT system (Promega, Madison, WI) in a 20 µl reaction mixture. A total of 2 µg RNA was used in the reaction and a random hexamer primer was used for the initiation of cDNA synthesis. After the RT procedure, the reaction mixture (cDNA) was used for PCR.

**Table 1:** Human gene specific-primers for RT-PCR.

Gene name	Accession No.	Forward primer(5′–3′)	Reverse primer (5′–3′)	Product size (bp)
GAPDH	J02642	AACAGCGACACCCACTCCTC	GGAGGGGAGATTCAGTGTGGT	258
KCa1.1	U11058	ACAACATCTCCCCCAACC	TCATCACCTTCTTTCCAATTC	310
KCa3.1	NM_002250	TGAGACGCCGAAAGCG	GCAGAGGAGTAAGAAGGTGGAA	187
KCa2.1	NM_170782	GAAGTTCCTCCAAGCTATCCA	TCTTTCCGTTCCCTGGTCT	498
Kv1.4	NM_002233	CCAGAGGAACCAGGAGTC	CCACAGATAGAGGCAAAGA	426
Kv1.5	NM_002234	CAGTTCCCCAACACACTCCT	CTGAACTCAGGCAGGGTCTC	410
Kv1.6	NM_002235	CCTGTCGCTGTTTCCG	ACCACCATTGTTTCCACC	456
Kv2.1	NM_004975	GAGCAGATGAACGAGGAGC	ACAGGGCAATGGTGGAGA	196
Kv3.1	NM_004976	CGAGGACGAGCTGGAGATG	CCTTGGCTGCCTTGGAG	479
Kv4.2	NM_012281	ACATGCAGAGCAAACGGAA	GGACTGTGACTTGAAGGACGA	220
Kv4.3	AF187963	GCCTCCGAACTAGGCTTTCT	CCCTGCGTTTATCAGCTCTC	310
Kir1.1	NM_153765	GGGACTTGCTCACATCG	CCACATTGCCAAATTCTAT	355
Kir2.1	NM_000891	ACTTCCACTCCATGTCCC	CTTTACTCTTCCCGTTCC	365
Kir2.2	BC027982	CCAAGAAGCGGGCACAGA	TGGGCGACACCAGAAAGAT	243
Kir2.3	NM_152868	CGGAGACCCCAAGACCCA	TGCTCAGGTTGGCGAAGT	340
Clcn2	NM_004366	AAGCGTGTCCGAATCTCC	ACCTCAGTGGTCTCCGTGT	368
Clcn3	NM_173872	CATAGGTCAAGCAGAGGGTC	TATTTCCGCAGCAACAGG	293
Nav1.1	NM_006920	GAGAACGACTTCGCAGATG	CACCAACCAAGGAAACCA	208
Nav1.2	NM_021007	CCCCTTCTACCCTCACATCT	ACACTGCTGAACTGCTCC	394
Nav1.3	NM_006922	AAAGAGCCGTGAGCATAG	ATCCCTCCACATTTGACA	432
Nav1.4	NM_000334	GTCATTCGCACCATCCTA	TCTCGCACTCAGACTTGTT	454
Nav1.5	NM_198056	ATGGACCCGTTTACTGACC	CCACTGAGTTCCCGATGAT	367
Nav1.6	NM_014191	TGCGGGAAAGTACCACTA	AGAAGGAGCCGAAGATGA	314
Nav1.7	NM_002977	AAAAGGCGTTGTAGTTCC	CAGTCATTGGGTGGTGTT	310
Nav1.8	NM_006514	AACTTCCGTCGCTTTACTC	GAAGGTCAGTTCGGGTCA	424
Nav1.9	NM_006514	TGATGACTGACCCGTTTA	ACAATGACCAGGACCACA	415

GAPDH, glyceraldehyde-3-phosphate dehydrogenase; KCa, Ca^2+^-activated K^+^ channel; Kv, voltage-gated K^+^ channel; Kir, inward rectifier K^+^ channel; Clcn Cl^−^ channel; Nav, voltage-gated Na^+^ channel.

PCR was performed with thermal cycling conditions of 94°C for 2 min followed by 35 cycles at 94°C for 45 s, 55–58°C for 45 s, and 72°C for 1 min using a Promega PCR kit and oligonucleotide primers as shown in Table 1. This was followed by a final extension at 72°C (10 min) to ensure complete product extension. The PCR products were electrophoresed through 1.5% agarose gels and visualized under a UV transilluminator (BioRad, Hercules, CA) after staining with ethidium bromide.

### Statistical analysis

Results are presented as means ± SEM. Paired and/or unpaired Student's *t*-tests were used as appropriate to evaluate the statistical significance of differences between two group means, and analysis of variance was used for multiple groups. Values of *P*<0.05 were considered to indicate statistical significance.

## Results

### Families of membrane ionic currents in human cardiac fibroblasts

**Figure 1:**
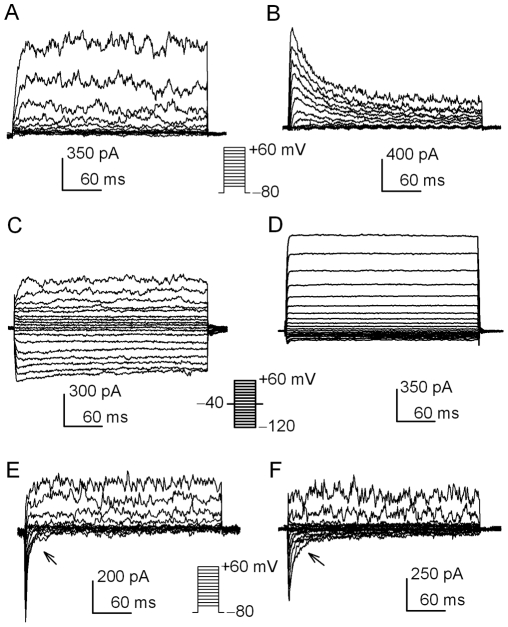
Families of membrane currents in human cardiac fibroblasts. *A.* Noisy current was activated at positive potential. Currents were elicited with the protocol shown in the *inset* (0.2 Hz). *B.* A transient outward current was activated in a human cardiac fibroblast by the same protocol as in A. *C.* A current with inward rectification activated by hyperpolarized potentials (*inset*) was co-present with the noisy current. *D.* Voltage-dependent current with outward rectification was recorded with the same protocol as in C. *E.* An inward current with fast inactivation activated by depolarization voltage steps (*inset*) was co-present with the noisy current. *F.* An inward current with slow inactivation (arrow) activated by the same protocol as in E was co-present with the noisy current.

Figure 1 illustrates the families of membrane currents recorded in human cardiac fibroblasts using a standard pipette solution. Five types of membrane currents were observed in human cardiac fibroblasts (in a total of 265 cells). One current was activated at depolarization voltages between −70 and +60 from a holding potential of −80 mV (0.2 Hz), and showed an outward current with noisy oscillation between +20 and +60 mV (Fig. 1A). These features suggest that this current is likely a big conductance Ca^2+^-activated K^+^ current (BK_Ca_) [15]. The noisy oscillatory BK_Ca_ was present with other currents in most (88%, 233 of 265) of fibroblasts. Another current activated by the same protocol was a transient outward current (Fig. 1B), and presented in 15% (40 of 265) of cells. Third current was an inward component activated by hyperpolarization voltage steps a holding potential of −40 mV and co-existed with the noisy oscillatory current activated by depolarization voltage steps. This inward component exhibited the properties similar to inward rectifier K^+^ current (I_Kir_) (Fig. 1C). I_Kir_ was observed in 24% (64 of 265) of cells. Fourth current was elicited by voltage steps between −120 and +60 from a holding potential of −40 mV, showing a very small inward component and a large outward current with outward rectification (Fig. 1D). This current was observed in 7% (19 of 265) of cells. Moreover, an inward current coexists with the oscillatory current in 61% (167 of 265) of human cardiac fibroblasts (Fig. 1E and 1F). Interestingly, the inward current exhibits either a fast inactivation (Fig. 1E) or a slow (Fig. 1F) inactivation.

### Ca^2+^-activated noisy oscillatory current

**Figure 2 pone-0007307-g002:**
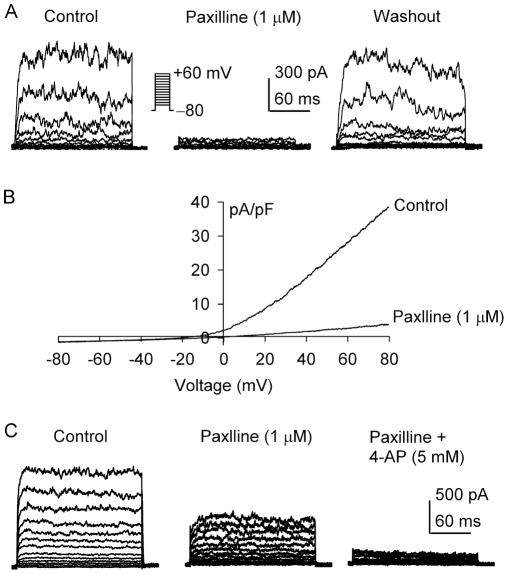
BK_Ca_ and IK_DR_ in human cardiac fibroblasts. *A.* Voltage-dependent current was reversibly suppressed by the BK_Ca_ blocker paxilline (1 µM). Currents were elicited by the voltage protocol as shown in the *inset*. *B.* Current-voltage (*I-V*) relationships of membrane current were recorded by a 2-s ramp protocol (−80 to +80 mV from a holding potential −40 mV) in a representative cell in the absence and presence of 1 µM paxilline. *C.* Membrane currents recorded in a typical experiment with the same voltage protocol as in A were partially inhibited by 1 µM paxilline. The remaining current was suppressed by co-application of paxilline and 5 mM 4-AP.

Figure 2A displays the noisy oscillatory BK_Ca_ reversibly suppressed by the BK_Ca_ blocker paxilline (1 µM, 5 min exposure) in a representative fibroblast. Current-voltage (*I-V*) curves recorded with a 2-s voltage ramp (−80 to +80 mV from a holding potential of −40 mV) in the absence of paxilline showed outward rectification (control) in another cell. The outwardly-rectifying current was remarkably reduced by paxilline (Fig. 2B). The current at +60 mV was reduced from 29.8±5.3 pA/pF of control to 3.9±2.1 pA/pF with 1 µM paxilline (n = 35, P<0.01 vs control).

We found that a paxilline-resistant current was present in a small population of human cardiac fibroblasts (14.2%, 5 of 35 cells). Figure 2C displays that paxilline (1 µM) partially suppressed the membrane current (+60 mV, to 8.9±1.6 pA/pF from 21.8.1±2.9, P<0.01); the remaining current was inhibited by 5 mM 4-aminopyridine (4-AP, to 2.1±1.1 pA/pF, n = 5, P<0.01) (Fig. 2C). This suggests that a 4-AP sensitive delayed rectifier K^+^ current (IK_DR_) is co-present with BK_Ca_ in these cells.

### Transient outward K^+^ current

**Figure 3:**
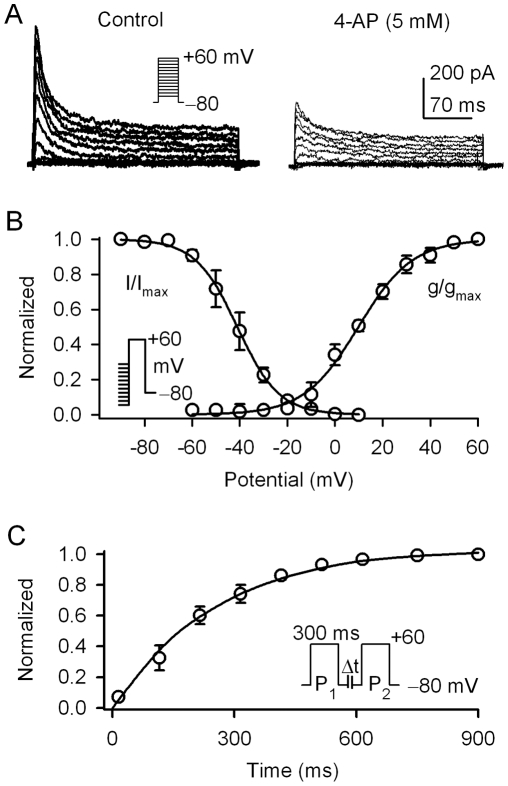
I_to_ in human cardiac fibroblasts. *A.* I_to_ traces recorded in a representative cell with the voltage protocol showed in the *inset* in the absence and presence of 5 mM 4-AP. *B.* Normalized mean values of voltage-dependent availability (I/I_max_) and activation conductance (g/g_max_) of I_to_ were fitted to the Boltzmann function: y = 1/{1+exp[(V_m_−V_0.5_)/S]}, where V_m_ is membrane potential, V_0.5_ is the estimated midpoint, and S is the slope factor. *C.* Normalized I_to_ (I_2_/I_1_) plotted vs. P_1_−P_2_ interval. The recovery curve was fitted to a mono-exponential function. The I_to_ was measured from the current peak to the ‘quasi’-steady-state level.

The transient outward K^+^ current I_to_ was present in 15% of cardiac fibroblasts. I_to_ in human cardiac myocytes was sensitive to inhibition by 4-AP [18], therefore we determined whether I_to_ in human fibroblasts could be decreased by 4-AP. Figure 3A shows the I_to_ traces recorded in a typical experiment in the absence and presence of 5 mM 4-AP. I_to_ (+60 mV) was substantially inhibited by 4-AP to 11.9±1.4 pA/pF from 36.5±2.6 pA/pF (n = 7, P<0.01).

Figure 3B illustrates the mean values of voltage-dependent activation (g/g_max_) and inactivation (availability, I/I_max_) of I_to_. The g/g_max_ was determined from the *I-V* relationship of each cell as previously described [19]. The I/I_max_ was determined with the protocol as shown in the left *inset* (with 1-s conditioning pulses from voltages between −100 and −10 mV followed by a 300-ms test pulse to +60 mV). Data were fitted to a Boltzmann distribution to obtain the half activation or availability voltage (V_0.5_) and the slope factor (S). The V_0.5_s of activation and availability of I_to_ were 11.2±0.4 mV (n = 7) and −40.6±1.5 mV (n = 9), and the S was 11.1±1.1 and −8.4±1.3, respectively.

Fig. 3C shows the time course of the mean values of I_to_ recovery from inactivation, determined with a paired-pulse protocol as shown in the *inset*. I_to_ recovery was complete within 900 ms and fitted to a mono-exponential function with time constant (τ) of 257.4±5.9 ms (n = 7). These properties of I_to_ in human cardiac fibroblasts, i.e. 4-AP sensitivity, voltage-dependent activation and availability, and recovery from inactivation, are similar to those observed in human cardiac myocytes [19] and mesenchymal stem cells [15], though there are differences in the values of the recovery time constant and the V_0.5_s of voltage-dependent activation and availability.

### Inward rectifier K^+^ current

**Figure 4:**
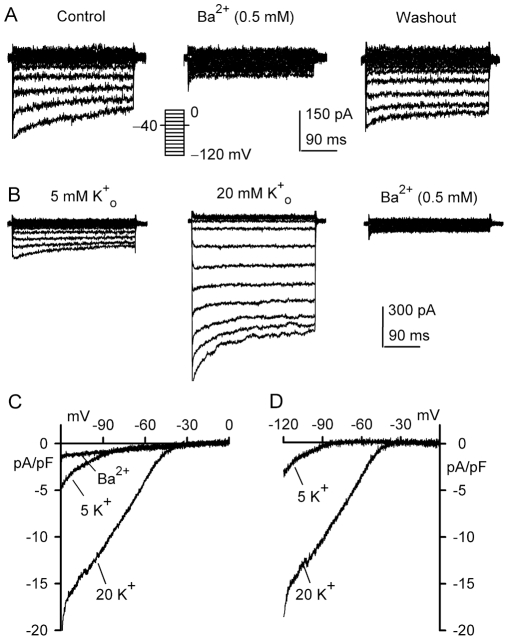
Effect of Ba^2+^ on membrane current in human cardiac fibroblasts. *A.* Voltage-dependent currents were reversibly inhibited by 0.5 mM BaCl_2_ in a representative cell. Currents were recorded with the protocol as shown in the *inset* (0.2 Hz). *B.* Voltage-dependent current recorded in another cell with voltage protocol shown in the *inset* of A was increased by elevating K^+^_o_ from 5 to 20 mM. Ba^2+^ (0.5 mM) remarkably suppressed the current. *C.* Left panel: *I-V* relationships of membrane currents recorded in a representative cell with a 2-s ramp protocol (−120 to 0 mV from a holding potential of −40 mV) in 5 mM K^+^_o_, 20 mM K^+^_o_, and after application of 0.5 mM Ba^2+^. Right panel: Ba^2+^-sensitive *I-V* relationships of the membrane current, typical of I_Kir_.

It is generally believed that inwardly-rectifying K^+^ channels are sensitive to inhibition by Ba^2+^ [20], therefore we determined the effect of Ba^2+^ on I_Kir_ in human cardiac fibroblasts. Figure 4 shows the current traces recorded in a representative cell with the voltage protocol as shown in the *inset* in the absence (control) and presence of Ba^2+^. Ba^2+^ (0.5 mM) reversibly reduced I_Kir_. Figure 4B displays that the increase of external K^+^ (K^+^_o_, from 5 to 20 mM) enhanced I_Kir_ conductance. Basal I_Kir_ and the high K^+^_o_-induced current were suppressed by Ba^2+^. Figure 4C illustrates the *I-V* relationships of I_Kir_ recorded in a representative cell with a 2-s ramp protocol (−120 to 0 mV from −40 mV) in solution containing 5 mM K^+^ (control) or 20 mM K^+^, and after application of 0.5 mM Ba^2+^ in bath solution. Ba^2+^ strongly inhibited I_Kir_. Ba^2+^-sensitive current was obtained by digitally subtracting currents before and after application of Ba^2+^ (Fig. 4D). The *I-V* relationships of Ba^2+^-sensitive I_Kir_ in 5 and 20 mM K^+^_o_ exhibited a strong inward rectification, typical of an inwardly-rectifying K^+^ current. Similar results were obtained in 5 other cells.

### Volume-sensitive chloride current in human cardiac fibroblasts

**Figure 5:**
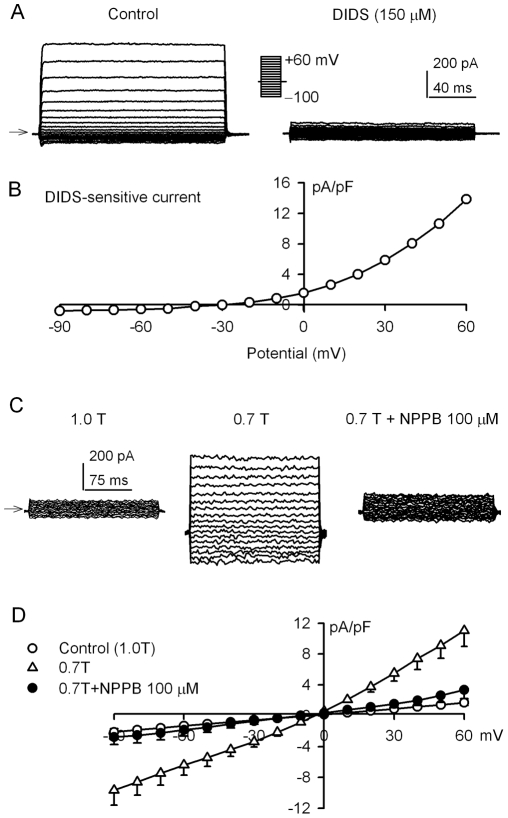
I_Cl_ in human cardiac fibroblasts. A. Voltage-dependent current was inhibited by the Cl^−^ channel blocker DIDS (150 µM). Current was elicited by the voltage steps as shown in the *inset* (0.2 Hz). *B.* *I-V* relation curve of DIDS-sensitive current obtained by subtracting currents before and after DIDS application in A. *C.* Voltage-dependent current recorded in a representative cells during control, after 20 min 0.7T exposure and application of 100 µM NPPB. *D.* *I-V* relationships for control current (1.0T), 0.7T and 0.7T with 100 µM NPPB. The 0.7T-induced current was significantly inhibited by NPPB at all test potentials (n* = *5, P<0.01). The arrows in the figure indicate the zero current level.

The current with outward rectification shown in Fig. 1D was insensitive to inhibition of K^+^ channel blockers including 5 mM tetraethylammonium (TEA), 5 mM 4-AP, or 0.5 mM Ba^2+^ (n = 4−6), suggesting that the outwardly-rectifying current is not carried by K^+^ ion. We then employed the Cl^−^ channel inhibitor DIDS to determine whether the current is carried by chloride ions. Figure 5A shows the current traces recorded in a representative cell with the protocol shown in the *inset*; DIDS (150 µM) suppressed the current. The *I-V* relationship (Fig. 5B) of the DIDS-sensitive current obtained by subtracting control currents by the current recorded after DIDS application displayed outward rectification and had a reversal potential at −35 mV, which is close to Cl^−^ equilibrium potential (*E*_Cl_, −46.8 mV). Similar results were obtained in a total of 6 cells. This result suggests that the recorded current under isotonic conditions is carried by Cl^−^ ions.

To investigate whether the Cl^−^ channel is volume sensitive in human cardiac fibroblasts, we employed a 0.7T tonic solution and recorded membrane current using a K^+^-free pipette solution, symmetrical Cl^−^ ion in pipette and bath medium as described in the Methods section. The membrane conductance was remarkably enhanced by exposure to 0.7T (20 min), and the increased current was highly suppressed by the Cl^−^ channel blocker NPPB (Fig. 5C). The *I-V* relationship of 0.7T-induced Cl^−^ current is linear under symmetrical Cl^−^ conditions Fig. 5D), similar to the previous report [21]. These results indicate that volume-sensitive Cl^−^ channel (I_Cl.vol_) is present in human cardiac fibroblasts.

### Inward Na^+^ currents in human cardiac fibroblasts

**Figure 6:**
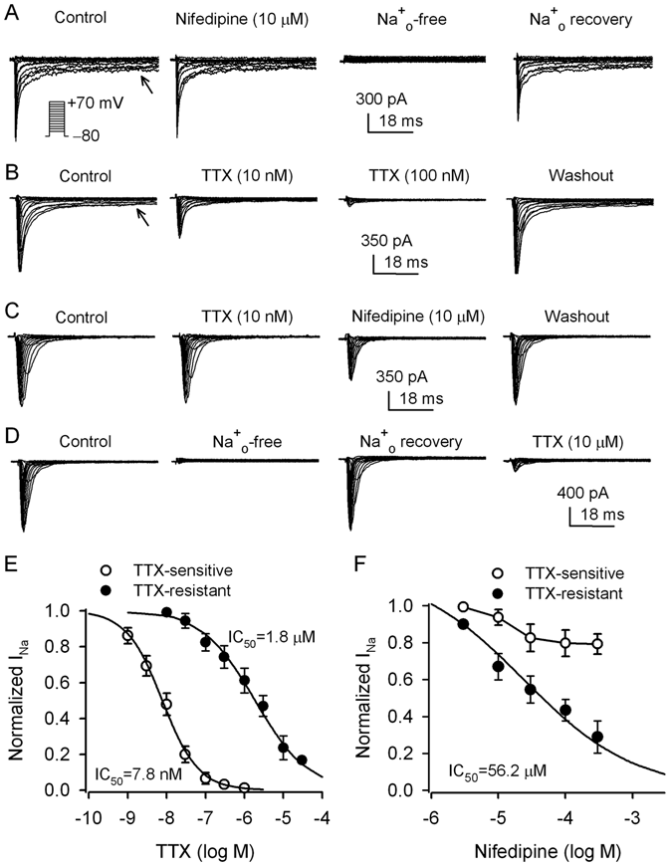
I_Na.TTX_ and I_Na.TTXR_ in human cardiac fibroblasts. *A.* An inward current with a persistent component (arrow) recorded in a representative cell under K^+^-free conditions using the voltage steps as shown in the *inset*. Nifedipine (10 µM) had no effect on the current, while the current disappeared when Na^+^_o_ was replaced with equimolar choline, and recovered as restoration of Na^+^_o_. *B.* Similar inward current with persistent component (arrow) recorded in another cell was highly sensitive to inhibition by low concentrations of TTX. *C.* An inward current with fast inactivation recorded using the same voltage protocol as shown in the *inset* of A. The current was not affected by 10 nM TTX, but reversibly inhibited by 10 µM nifedipine. *D.* Similar current recorded in another cell disappeared with Na^+^_o_ removal, and recovered as restoration of Na^+^_o_. The current was suppressed by a high concentration of TTX (10 µM). *E.* Concentration-dependent response of two types of inward currents to TTX. The data were fitted to the Hill equation: E = E_max_/[1+(IC_50_/C)^b^], where E is the percentage inhibition of current at concentration C, E_max_ is the maximum inhibition, IC_50_ is the concentration for a half inhibitory effect, and b is the Hill coefficient. The IC_50_ of TTX for inhibiting TTX-sensitive I_Na_ was 7.8 nM (n = 5−9 for each concentration), the Hill coefficient was 0.94. The IC_50_ of TTX for inhibiting TTX-resistant I_Na_ was 1.8 µM (n = 6−9 cell for each concentration), the Hill coefficient was 0.58. *F.* Concentration-dependent relationships of I_Na.TTX_ and I_Na.TTXR_ to nifedipine. The IC_50_ of nifedipine for inhibiting I_Na.TTXR_ was 56.2 µM (n = 4−7 cells for each concentration) with a Hill coefficient of 0.59.

The depolarization-elicited inward currents (Fig. 1E and 1F) were studied under K^+^-free conditions. Figure 6 illustrates two types of inward currents recorded in human cardiac fibroblasts with voltage steps (50 ms) to between −60 and +70 mV from −80 mV (*inset*) in 10-mV increments at 0.2 Hz. One of these currents exhibited an incomplete inactivation (or a persistent component) during 50 ms depolarization (control of Fig. 6A, 6B), similar to L-type Ca^2+^ current (I_Ca.L_) in human cardiac myocytes [22]. However, this current was insensitive to inhibition by a high concentration of the I_Ca.L_ blocker nifedipine (10 µM), in contrast with human cardiac I_Ca.L_, which is fully suppressed by nifedipine [22]. Interestingly, the current was abolished by replacing bath Na^+^ (Na^+^_o_) with equimolar choline, and recovered upon restoration of Na^+^_o_ (Fig. 6A, n = 6). In addition, this current is sensitive to inhibition by 10 and 100 nM tetrodotoxin (TTX), and the effect was reversed by washout (n = 6). These results suggest that this inward current is likely a TTX-sensitive I_Na_ (I_Na.TTX_) with a persistent component.

Another inward current exhibited a complete inactivation (control of Fig. 6C & 6D). This current had no response to 10 nM TTX; however, nifedipine (10 µM) reversibly reduced the current (Fig. 6C, n = 7). Replacement of Na^+^_o_ with equimolar choline reversibly abolished this inward current, and the current required a high concentration (10 µM) of TTX for a substantial suppression (Fig. 6D, n = 6). These results suggest that this inward current is likely a TTX-resistant Na^+^ current (I_Na.TTXR_).

The concentration-dependent inhibitory effects of TTX on I_Na.TTX_ and I_Na.TTXR_ at 0 mV are illustrated in Fig. 6E. The IC_50_ (50% inhibitory concentration) of TTX for inhibiting I_Na.TTX_ was 7.8 nM with a coefficient of 0.94, while the IC_50_ of TTX for inhibiting I_Na.TTXR_ was 1.8 µM with a Hill coefficient of 0.58. The I_Ca.L_ blocker nifedipine had no significant inhibitory effect on I_Na.TTX_, whereas it inhibited I_Na.TTXR_ with an IC_50_ of 56.2 µM and a Hill coefficient of 0.59 (Fig. 6F).

**Figure 7:**
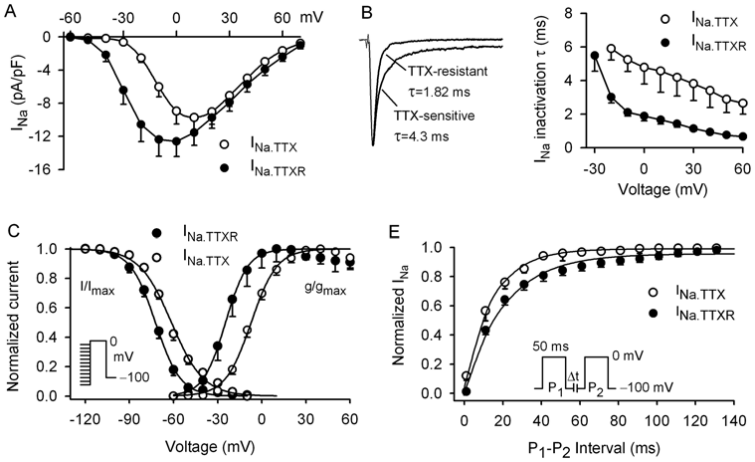
Kinetics of I_Na.TTX_ and I_Na.TTXR_. *A.* Mean values of *I-V* relationships of I_Na.TTX_ and I_Na.TTXR_. *B.* Left panel: inactivation time course of representative I_Na_ traces (at 0 mV) was fitted to a monoexponential function with time constant (τ) shown, 4.3 ms for I_Na.TTX_ and 1.82 ms for I_Na.TTXR_. Right panel: mean values of voltage dependence of inactivation of I_Na.TTX_ (n = 8) and I_Na.TTXR_ (n = 10). P<0.05 or P<0.01 at −20 to +60 mV. *C.* Voltage-dependent availability (I/I_max_) of I_Na_ was determined with the protocol as shown in the left *inset* (with 1-s conditioning pulses from voltages between −120 and −10 mV then a 50-ms test pulse to 0 mV). Curves of I/I_max_ and activation conductance (g/g_max_) were fitted to a Boltzmann equation. *E.* Recovery curves of I_Na.TTX_ and I_Na.TTXR_ from inactivation were fitted to a monoexponential function.

The *I-V* relationships for the peak current of I_Na.TTX_ and I_Na.TTXR_ are illustrated in Fig. 7A. I_Na.TTX_ had a threshold potential of −40 mV and peaked at +10 mV, while I_Na.TTXR_ had a threshold potential of −50 mV and peaked at 0 mV. Inactivation of I_Na.TTX_ and I_Na.TTXR_ was fitted to a monoexponential function with time constant (τ) as shown in the left panel of Fig. 7B. The inactivation process of I_Na.TTX_ was slower than that of I_Na.TTXR_ (Fig. 7B, n = 12, P<0.01 at −20 to +60 mV).

Figure 7C illustrates the mean values of the steady-state voltage dependent activation (g/g_max_) and inactivation (availability, I/I_max_) for both I_Na.TTX_ and I_Na.TTXR_. The g/g_max_ was determined from the *I-V* relationships of each cell in Fig. 7A as previously described [23]. The I/I_max_ was determined with the protocol as shown in the left *inset* (with 1-s conditioning pulses from voltages between −120 and −10 mV then to a 50-ms test pulse to 0 mV). Data were fitted to a Boltzmann equation. The V_0.5_s of g/g_max_ and I/I_max_ for I_Na.TTX_ were −7.2±1.1 mV (n = 9) and −61.4±1.6 mV (n = 10), and the S was 8.7±1.2 and −10.8±1.2, respectively. While the V_0.5_s of g/g_max_ and I/I_max_ for I_Na.TTXR_ were −24.7±1.4 (n = 7) and −72.3±1.5 (n = 9) mV, and the S was 7.5±1.1 and −8.5±1.3, respectively. The V_0.5_s of g/g_max_ and I/I_max_ were more positive in I_Na.TTX_ than those in I_Na.TTXR_ (P<0.01).

Figure 7E shows the time course of mean values of recovery of I_Na.TTX_ or I_Na.TTXR_ from inactivation, which was determined using a paired-pulse protocol shown in the *inset* as described previously [23]. The recovery of I_Na.TTX_ and I_Na.TTXR_ from inactivation was complete within 150 ms, and the curves were fitted to a mono-exponential function. The time constant (τ) was 14.3±2.1 ms for I_Na.TTX_ (n = 11) and 21.4±2.9 ms for I_Na.TTXR_ (n = 9). The recovery of I_Na.TTXR_ from inactivation was slower than that of I_Na.TTX_ (P<0.05). These results indicate that two types of Na^+^ channels with distinct TTX-sensitivity and kinetics are present in human cardiac fibroblasts.

### Messenger RNAs of functional ion channels

**Figure 8:**
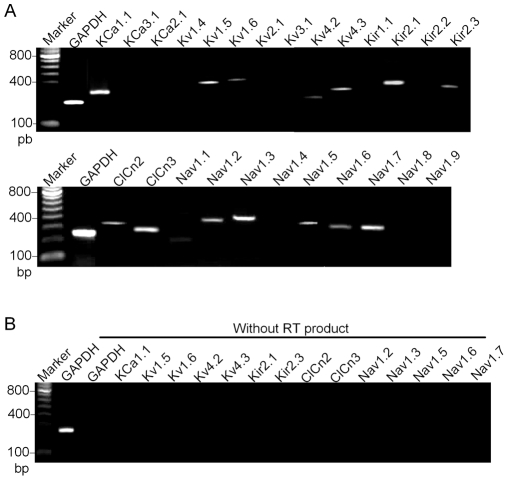
RT-PCR for detecting ion channels expressed in human cardiac fibroblasts. *A.* Images of RT-PCR products corresponding to significant gene expression of KCa1.1 (BK_Ca_), Kv1.5 (IK_DR_), Kv4.3 (I_to_), and Kir2.1 (I_Kir_) and Clcn3 (I_Cl.vol_), and Na_V_1.2, Na_V_1.3, Na_V_1.5, Na_V_1.6 and Na_V_1.7 in human cardiac fibroblasts. A weak expression of Kv4.2, Kir2.3, Clcn2 and Na_V_1.1 was also found in human cardiac fibroblasts. *B.* No significant bands were observed in the PCR experiment when RT product was replaced by total RNA.

To explore the molecular identities of the functional ionic currents, we examined gene expression of various ionic channels in human cardiac fibroblasts with RT-PCR using the specific primers targeting human genes for KCa, Kv, Kir, Clcn, and Na_V_ channel families as shown in Table 1. Figure 8A displays the significant gene expression of KCa1.1 (responsible for BK_Ca_), Kv1.5, Kv1.6 (responsible for IK_DR_), Kv4.2, Kv4.3 (responsible for I_to_), Kir2.1, Kir2.3 (for I_Kir_), Clcn3 (for I_Cl.vol_), Na_V_1.2, Na_V_1.3, Na_V_1.6 and Na_V_1.7 (for I_Na.TTX_), and Na_V_1.5 (for I_Na.TTXR_) in human cardiac fibroblasts. In addition, Clcn2 was also significantly expressed in human cardiac fibroblasts. When RNA was directly amplified by PCR without reverse transcription, the bands for these positive genes disappeared (Fig. 8B), suggesting that the genes detected were not false-positive signals from genomic DNA contamination.

## Discussion

In the present study, we have demonstrated that multiple ionic currents (BK_Ca_, IK_DR_, I_to_, I_Kir_, I_Cl.vol_, and I_Na.TTX_ and I_Na.TTXR_) are present in human cardiac fibroblasts. BK_Ca_ was inhibited by paxilline, IK_DR_ and I_to_ were inhibited by 4-AP. I_Kir_ was blocked by Ba^2+^ and I_Cl.vol_ was inhibited by DIDS or NPPB, while I_Na.TTX_ and I_Na.TTXR_ were suppressed by different concentrations of TTX. The channel genes corresponding to the functional currents (KCa1.1 for BK_Ca_, Kv1.5/Kv1.6 for IK_DR_, Kv4.2/Kv4.3 for I_to_, Kir2.1/Kir2.3 for I_Kir_, Clcn3 for I_Cl.vol_, Na_V_1.2/Na_V_1.3/Na_V_1.6/Na_V_1.7 for I_Na.TTX_, and Na_V_1.5 for I_Na.TTXR_) were confirmed by RT-PCR.

Recent studies demonstrated that an inward rectifier K^+^ current (I_Kir_), a delayed rectifier K^+^ current (IK_DR_), and a non-selective cation channel current were present in rat ventricular fibroblasts [11]–[13]. Only BK_Ca_ was described in human cardiac fibroblasts [14]. The present study provides novel information that multiple ion channels are heterogeneously expressed in human cardiac fibroblasts. In addition to BK_Ca_ as previously reported by Wang and colleagues [14], IK_DR_, I_to_, I_Kir_, I_Cl.vol_, I_Na.TTX_, and I_Na.TTXR_ were present with BK_Ca_ in different populations of human cardiac fibroblasts (Fig. 1). These currents have different distribution and properties compared to those in human cardiomyocytes [18], [24]–[27].

Several K^+^ currents have been reported in myocytes from human hearts. They include 4-AP-sensitive I_to_ (encoded by Kv1.4/Kv4.3) [28] in atrial and ventricular myocytes [18], [26], 4-AP sensitive ultra-rapid delayed rectifier K^+^ current (I_Kur_, encoded by Kv1.5) in atrial myocytes [29], inward rectifier K^+^ current (I_K1_, encoded by Kir2.1/Kir2.3) [24], [28], [30], and rapidly and slowly-activated delayed rectifier K^+^ currents (I_Kr_ and I_Ks_) [25]. However, I_to_ (likely encoded by Kv4.2/Kv4.3) and IK_DR_ (likely encoded by Kv1.5/Kv1.6) were present only in a small population of human cardiac fibroblasts (15% and 14%, respectively) (Figs. 1–3). In addition, Ba^2+^-sensitive inward rectifier K^+^ current (likely encoded by Kir2.1/Kir2.3) with a small amplitude was present in 24% human cardiac fibroblasts, not like in human cardiomyocytes where I_K1_ is detected in each cell [24], [30]. It is interesting to note that BK_Ca_ was present in most (88%) human cardiac fibroblasts; however, this current has not been identified in human cardiomyocytes. The different distribution of K^+^ currents implies the various functions of these channels in these two types of heart cells.

Earlier studies have demonstrated that I_Cl.vol_ are present in human cardiac myocytes [31], [32], and the current is only recorded when the hypotonic insult is applied [31], [32]. Nonetheless, I_Cl.vol_ is recorded in a small population (7%) of human cardiac fibroblasts without hypotonic exposure (Fig. 1), and it is activated in almost all fibroblasts with hypotonic exposure (Fig. 5). I_Cl.vol_ is believed to play a role in arrhythmogenesis, myocardial injury, preconditioning, and apoptosis of myocytes [33]. Nonetheless, physiological function of I_Cl.vol_ in human cardiac fibroblasts remains to be studied in the future.

It is well recognized that I_Na_ channels expressed in cardiomyocytes (mainly encoded by Na_V_1.5) play an important role in controlling excitation-contraction and impulse conduction in the hearts. I_Na_ has been also found to participate in regulating sinus node pacemaker function [34]. In the present study, we found that I_Na_ was expressed in most (61%) human cardiac fibroblasts (Fig. 1). The I_Na.TTX_ in human cardiac fibroblasts (Figs. 6 & 7) shares some properties with neuronal I_Na_, e.g. a transient inward current followed by a persistent component, sensitive to inhibition by nanomolar TTX, and likely encoded by Na_V_1.2, Na_V_1.3, Na_V_1.6, and Na_V_1.7 [35], [36].

The I_Na.TTXR_ in human cardiac fibroblasts (Figs. 6 & 7) shares some features with I_Na_ in cardiomyocytes (e.g. inhibited by micromolar TTX and encoded by Na_V_1.5) [35], [37]. Some properties of I_Na.TTXR_ in human cardiac fibroblasts are not identical to those of I_Na_ in human cardiomyocytes [27], [37], [38], e.g. more positive V_0.5_s of activation (−25 mV vs −39 mV) and availability (−72 mV vs −95 mV) and more positive peak current potential (0 mV vs −35 mV), compared to I_Na_ in human cardiomyocytes [27], [37]. In addition, I_Na.TTXR_ in cardiac fibroblasts, as Na_V_1.5-encoded I_Na.TTXR_ in gastric epithelial cells [39], was inhibited by high concentrations of the I_Ca.L_ blocker nifedipine (Fig. 6). Nonetheless, no report is available in the literature regarding the information whether I_Na_ of cardiomyocytes is sensitive to a high concentration of nifedipine. Moreover, it is unknown how I_Na.TTX_ and I_Na.TTXR_ participate in cellular function of cardiac fibroblasts.

It has been recognized that cardiac fibroblasts are electrically unexcitable, but they contribute to the electrophysiology of myocytes in various ways, such as electrical coupling of fibroblasts and myocytes [40]. The electrical coupling between fibroblasts and myocytes was observed at cellular and tissue level as well as in cell cultures [5], [8], [40]–[42]. Coupling between fibroblasts and myocytes was demonstrated to be via Cx43 gap junctions in sheep ventricles and Cx45 in rabbit sinoatrial node cells [4], [6] and in sheep ventricular scars [43]. The cardiac fibroblasts are therefore believed to maintain electrical contact with myocytes. Our results of multiple ion channels in human cardiac fibroblasts likely provide a basis for understanding of the potential contribution of these ion channels to fibroblast-myocytes electrical coupling under physiological conditions, and also for future studies on the potential mechanism how cardiac fibroblasts participate in regulating cardiac electrophysiology.

In proliferative cells, ion channels play a role in cell cycle progression [44], [45]. The activity of BK_Ca_ (i.e. KCa1.1) channels was regulated by the spontaneous Ca^2+^ oscillations, resulting in fluctuations of membrane currents and potentials. BK_Ca_ was reported to play a role in regulating proliferation of human preadipocytes [46], endothelial cells [47], and breast cancer cells [48]. I_Kir_ was found to participate in regulating the proliferation of human hematopoietic progenitor cells [49]. Although the underlying mechanisms of ion channels in cell proliferation regulation remain elusive, the involvement of K^+^ channels in cell proliferation was well established [44], [45], [49]. Further exploration is required to find out whether these ion channels contribute to human cardiac fibroblast proliferation.

Clcn3 channel is regarded as one of the candidate channels for volume regulated anion channels and has been shown to play an important role in cell proliferation and apoptosis [45]. Blockade or disruption of Clcn3 channel resulted in arrest of cell cycle and prevention of cell proliferation in several cell types [21], [50]. The present observation demonstrated that functional chloride current encoded by Clcn3, sensitive to cell volume, was observed in human cardiac fibroblasts (Fig. 5). Whether this I_Cl_ current would contribute to human cardiac fibroblast proliferation remains to be studied in the future.

One of limitations of the present study was that ion channels, BK_Ca_, I_to_, I_Kir_ and I_Cl.vol_, and I_Na.TTX_, and I_Na.TTXR_, were heterogeneously expressed within the same species of cultured human cardiac fibroblasts. This could result from heterogeneous cell population of the fibroblasts. An earlier study demonstrated that myofibroblast could differentiate from fibroblasts when plated at low density and could revert back to fibroblasts at higher density [51]. Consequently, a subpopulation of human cardiac fibroblasts may display different patterns of ion channel expression.

In summary, the present study provides the first information that multiple ion channel currents are present in cultured human cardiac fibroblasts, the patterns and properties of these ion channel currents differ from those observed in human cardiac myocytes. The information obtained form the present study provides a basis for future study how ion channels participate in regulating cardiac electrophysiology.
